# Michigan Men’s diabetes project (MenD): protocol for a peer leader diabetes self-management education and support intervention

**DOI:** 10.1186/s12889-021-10613-2

**Published:** 2021-03-22

**Authors:** Jaclynn Hawkins, Katherine Kloss, Martha Funnell, Robin Nwankwo, Claudia Schwenzer, Fonda Smith, Gretchen Piatt

**Affiliations:** 1 University of Michigan, School of Social Work, 1080 S. University, Ann Arbor, MI 48109 USA; 2 Department of Learning Health Sciences, University of Michigan, 1111 E. Catherine, Ann Arbor, MI 48109 USA

**Keywords:** Diabetes self-management, Gender, Peer leaders, Men, Diabetes social support

## Abstract

**Background:**

Black men are more likely to be diagnosed with type 2 diabetes (T2D) compared to non-Hispanic White men, and this disparity increases among men over the age of 55. A growing body of literature demonstrates the critical role of gender in the management of health behaviors such as T2D and shows that male gender norms can conflict with healthy behaviors. These studies suggest that tailoring diabetes self-management interventions to address the needs of Black men may be critical to helping them to achieve optimal health outcomes. Further, our own research on Blacks with T2D found gender disparities in participation in diabetes interventions, with males participating at significantly lower rates than females. Peer leaders are trained lay individuals who are used to provide ongoing diabetes self-management support to people with diabetes, particularly in minority communities. However, despite studies showing that diabetes management interventions using peer leaders have been successful, the majority of peer leaders as well as the participants in those studies are women. The limited studies to date suggest that Black men with T2D prefer peer-led, male-to-male T2D programs, however, this research consists primarily of nonrandomized, small sample feasibility studies calling for additional studies to establish the efficacy of these approaches. The proposed study will develop and preliminarily validate the effectiveness of an adapted peer leader diabetes self-management support (PLDSMS) intervention designed to improve diabetes-related lifestyle and self-management behaviors in Black men (over 55) with T2D.

**Method:**

We propose to tailor an existing intervention by 1) our using male peers and 2) modifying the peer leader training content to focus on material appropriate for men. The proposed study includes a developmental phase (development of the intervention with expert feedback, followed by feasibility testing with Black men) and a validation phase [randomized clinical trial (RCT)].

**Discussion:**

If successful, this study will lead to the development and dissemination of an intervention that will address the unique needs of Black men with T2D, helping them to achieve optimal diabetes self-management and health outcomes.

**Trial registration:**

Registered at ClinicalTrials.gov with an ID NCT04760444 on February 17, 2021

## Background and rationale

Diabetes is the 7th leading cause of death in both the United States [1] and in Michigan [2] and the 6th leading cause of death in Detroit. Diabetes is highly prevalent in Black men and they are more likely to be diagnosed with diabetes compared to non-Hispanic White men [2]. In addition, they are disproportionately more likely to have poorly managed blood glucose levels resulting in more complications than their non-Hispanic White counterparts [3].

One factor that may account for diabetes outcomes is differences in diabetes management behaviors among Black men as compared to non-Hispanic White men [4]. Studies suggest that Black men in general are less likely to adhere to diabetes self-management regimens [5, 6]. Although studies of older Black men are more limited, those that exist suggest that they are even less likely to engage in diabetes self-management behaviors (e.g. checking blood glucose levels daily) [5]. Black men are also more likely to engage in behaviors such as suboptimal diet that increase their risk for diabetes-related complications than their non-Hispanic White peers [7, 8]. Black men may also face unique barriers that interfere with healthy behaviors including a lack of social support, negative patient-provider relationships, cost and long work hours [8–10]. While existing research clearly shows that older Black men are at elevated risk for poor diabetes management, they are also less likely to participate in diabetes management intervention research studies [11]. Specifically, the percentage of male participants was typically less than 15%. This is also consistent with the finding that men are more likely to drop out of diabetes self-management intervention trials [8, 9, 12].

A growing body of literature also demonstrates the critical role of gender in the management of health behaviors and shows that male gender norms can conflict with both healthy behaviors and healthcare engagement [8, 9, 13]. While sex refers to biological differences (e.g. chromosomes and sex organs, gender describes the characteristics that a society or culture delineates as masculine or feminine. Among Black men specifically, the need to exhibit toughness, confidence, suppression of emotions and independence/control served as barriers to both accepting advice from care providers and accepting social support offered by family and community networks [13]. In a recent study, Black men reported encountering significant difficulty in accepting a caring environment, such as emotional support, as this was perceived as feminized behavior, which in turn served as a barrier to seeking care [13]. Gender-related norms among Black men also include “superman syndrome” or the ability to maintain their health without the assistance of health care providers [14, 15]. Recommendations for gender-specific programming have included using male leaders to reduce stigma and embarrassment related to health care seeking, improve communication with health care providers and address specific beliefs that undermine health [6, 16].

Peer leaders have been defined as trained lay individuals used to improve diabetes self-management in minority communities through provision of diabetes education, assistance with goal setting, problem solving, and social and emotional support [16–21]. In this context, peer leaders shared cultural identity and community ties are a critical component in improving diabetes management among minorities with diabetes [16–21]. However, despite studies showing that interventions using peer leader models have been successful in improving diabetes self-management and clinical outcomes (e.g. A1C) among minority populations, these interventions have not been adapted to meet the needs of older Black men. In addition, the majority of both participants and peer leaders are female in these studies. Matching lay leaders for the race and gender of patients may be one way to increase the likelihood that both intervention content and messaging are appropriate and result in improved outcomes [11].

While there is a paucity of research using Black male peer leaders for Black men with diabetes, male lay helpers have been used in other areas of health care to provide Black men with health education and support [22]. These interventions have included prevention of chronic illness (e.g. screening for hypertension) [23, 24]. However, this research is limited and additional work needs to be done to establish the efficacy of these approaches [23, 24] to assess whether use of male peers can improve health outcomes among Black men with diabetes.

Our work and that of others suggest that tailoring DSME/S interventions to address the needs of Black men is critical to helping them to lead healthier lives [4, 5]. The National Standards for Diabetes Care [25] and the National Standards for Diabetes Self-Management Education and Support [26] (DSMES) emphasize the importance of providing initial DSME and on-going diabetes self-management support (DSMS) to assist people with diabetes in maintaining effective self-management. DSMS interventions, including those delivered by professionals and non-professionals (e.g. peer leaders), improved A1C and self-management outcomes compared to control groups [27–36]. Piatt et al. tested the use of a similar, 12 session, 15 month intervention that combined DSME delivered by a diabetes educator with DSMS provided by peer leaders (PL) in 9 Black churches in metro-Detroit [36]. Intervention completion was high (96.6%). Twelve Peer Leaders (PL) (*n* = 12) were trained to use goal setting, skills development, and group facilitation. Following professionally delivered DSME, 6 monthly DSMS groups were facilitated by 12 PL (mean age: 61.9 years, 100% Black, 25% male), followed by an additional 6 months of ongoing support to assess the logistical feasibility of sustaining DSMS efforts [36]. In addition, other research suggests that Black men with diabetes prefer peer-led, male-to-male interventions [22, 23]. Use of male leaders to promote health behavior change is also consistent with approaches used for other chronic illnesses, such as HIV [22]. Despite this fact, the majority of peer leaders and participants in DSMS studies are women [11], which creates a critical gap in our understanding of the utility of DSMS among men.

As previously noted, there is a dearth of efficacious diabetes management interventions for Black men. While the effectiveness of peer-led education and support interventions are effective in the short-term, Black men, in particular, are less likely to participate and are at a higher risk of drop-out in these studies [11]. The proposed research is innovative, because 1) DSMS interventions to date have not specifically targeted Black men, and 2) peer-led DSMS interventions have not matched peer leaders based on the gender of intervention participants nor tailored the intervention content to meet the specific needs of men. We will develop training for our male peer leaders to specifically encourage conversations regarding beliefs that affect men’s health and to allow modeling of alternative views and perspectives that allow for successful disease management to be framed as competence and strength. In this proposal, the primary intervention component is not focused on DSME. Instead, the proposed study aims to assess the effectiveness of adapted DSMS, following DSME, over a 16-week period using a peer-led approach to DSMS. Additionally, this study will deliver important new information that builds on our previous work [28–40], in order to move the field forward in understanding what it takes to adapt and implement effective peer-led DSMS programming for Black men while also utilizing community resources (i.e. peer leaders) and infrastructures.

## Methods/design

### Objectives

The objective of this proposal is to adapt and evaluate the preliminary efficacy of a peer-led DSMS (PLDSMS) intervention for Black men with type 2 diabetes. To accomplish this objective, we will tailor an existing peer-led DSMS intervention, Praise [36], by 1) using male peer leaders as interventionists and 2) modifying the intervention content to focus on messaging appropriate for Black men.

The proposed study includes a developmental phase (adaptation of the intervention with stakeholder feedback, followed by feasibility testing with Black men) and a validation phase [randomized controlled trial (RCT)].

#### Phase 1: intervention adaptation

Adapt the Praise intervention for Black men with diabetes. Adaptation and refinement will involve conducting 10–15 semi-structured interviews to assess barriers and facilitators to diabetes self-management and to evaluate the feasibility and acceptability of intervention materials.

#### Phase 2: RCT

Conduct an RCT of the adapted intervention to evaluate participant recruitment and retention rates, treatment and intervention satisfaction and estimate intervention effect sizes on our primary outcomes (i.e., A1C and self-management behaviors) as well as on secondary outcomes (i.e., diabetes social support and diabetes-related distress).

The RCT will be conducted among *N* = 64 Black male adult residents of Detroit, MI. Sixty men will be randomized to a control group or to the tailored PLDSMS and four men will be recruited and trained to function as peer leaders. We hypothesize that 1) participants in the adapted PLDSMS approach will have improved outcomes over the control group, and 2) a participants in the PLDSMS will achieve DSME skills at significantly higher levels than participants in the control group.

The primary outcome will be change in A1C and self-management behaviors measured through completion of the Perceived Diabetes Self-Management Scale, a self-report questionnaire measuring a broad range of diabetes self-management behaviors, post intervention and at 3-month follow up. Secondary outcomes will be BMI, blood pressure, quality of life, diabetes related-distress, diabetes social support, and adherence to gender norms.

### Study setting

All DSME/S sessions will be held virtually via a Health Insurance Portability and Accountability Act (HIPPA) compliant virtual platform, Zoom [40] for Health at U-M. Baseline, post-treatment and follow-up self-report questionnaire will be completed telephonically or virtually.

Biometric data will be collected at a local senior center non-profit organization that provides comprehensive services to a diverse population of more than 2000 seniors living throughout metropolitan Detroit. In each location, rooms with doors and telephones will be available to facilitate privacy, confidentiality, and safety of assessments. This data will be collected at baseline, post-treatment, and follow-up.

### Eligibility criteria

#### Inclusion criteria

Participants will be 64 Black/African American men ages 55 years or older and meet the inclusion criteria listed below. Our study focuses on older Black men because: 1) the age incidence rate of diabetes onset is highest among older adults and 2) Black men are disproportionately more likely to receive a diabetes diagnosis at later stages of the disease, compared to Black women and non-Hispanic white men.

Our inclusion criteria include males age 55 or older who are Black/African American, and who’ve had a diagnosis of type 2 diabetes for a six-month duration or longer.

During the Phase II RCT we will individually randomize each participant with a 50/50 randomization scheme to either the peer led DSMS or a control group. Four of the participants will attend 30- h of training and function as peer leaders during the intervention DSMES.

We considered restricting eligibility to a higher-risk population of participants with A1C ≥ 8%. Preliminary data suggest that over 50% of the proposed study sample will have an A1C ≥ 8%. Focusing on all older Black men with diabetes allows us to cast a wide net for secondary prevention and public health impact. Persons who meet eligibility criteria will be invited to participate in the baseline screening assessment. While we have chosen the above eligibility criteria based on previous work, we will make adjustments to the future, larger trial, based on results and feedback from our proposed study.

#### Exclusion criteria

Our exclusion criteria include 1) non-ambulatory, serious health conditions (severe symptomatic heart disease, visual impairment, renal failure, and peripheral neuropathy); 2) psychiatric illness (severity requiring hospitalization); and 3) serious diabetes complications (e.g., blindness) that would impede meaningful participation.

### Interventions

#### Intervention description

##### Phase 1: development phase

We will tailor the existing intervention from Piatt et al’s^38^ previous peer leader trial (Praise) and add additional components to better address the needs of Black men with diabetes. Phase I will be guided by the Theoretical Domains Framework (TDF) a consolidated theoretical framework identifying 14 domains related to behavior change [41]. The TDF provides a lens for identifying cognitive, affective, social, and environmental influences on behaviors, which in turn inform behavior change targets for implementation interventions. In particular, we will conduct 10–15 semi-structured interviews groups to assess barriers and facilitators to diabetes self-management and to evaluate the feasibility and acceptability of intervention materials. The TDF will enable the interview findings to be linked back to theory to facilitate an understanding of the behavior change processes that are barriers or facilitators to implementation. Theory-driven refinements will then be made to optimize the intervention content and processes (i.e. domain-specific beliefs informed changes and improvements to intervention components) that are congruent with intervention components. The TDF will be used to identify domain-specific issues, specifically for Black men, with the DSMS intervention materials (e.g. knowledge/skills/support gaps). The interviews will also identify themes to be incorporated into intervention content and training manuals that relate to Black men with diabetes. After materials are revised, they will be reviewed by the study team. We will then make adaptations to the training manuals and intervention content based on our findings using the TDF domains. Specifically, participant recruitment and educational materials will be adapted for gender. Intervention materials have already been adapted to account for language, culture, health literacy and urban poverty contexts.

In a future, longer term and adequately powered trial, two versions of the manuals will be more rigorously adapted and tested based on our study findings: one for interventionists and one for patients. In the DSMS protocol, standardized treatment goals and homework assignments will be adapted to incorporate local references, cultural values and familiar situations. Although module adaptations cannot be prespecified, based on the existing literature we will develop a DSMS module that will focus on teaching male participants how to optimize clinic appointments with their diabetes care providers (i.e. being assertive, articulating concerns, and asking specific questions).

##### Phase 2

This study will include a 6-month randomized control trial (RCT) of virtual diabetes self-management education and support (DSME/S) with peer leaders to evaluate participant recruitment and retention rates, treatment and intervention satisfaction and estimate intervention effect sizes on our primary outcomes of self-management behaviors as well as on secondary outcomes such as glycemic control (A1C), diabetes social support, diabetes-related distress, and adherence to gender norms. This data will be collected at baseline, 3 months (treatment termination), and 6 months (3 months post treatment termination) at a senior center in Detroit, telephonically, or virtually. Participants will be randomized to the virtual peer leader DSMES or a control group. All virtual DSME/S sessions will be held on the ZOOM platform. The baseline assessment and intervention period will consist of 16 weeks. In the first week participants will complete baseline questionnaires, interviews telephonically or virtually.

##### Intervention (peer leader DSMS *n* = 30)

Participants randomized to the peer led DSMS group will receive 6100-min weekly sessions of diabetes self-management education (DSME) delivered by a certified diabetes care and education specialists (CDCES) and co-facilitated by a peer leader (PL) delivered via CDCES-led DSME was chosen to ensure consistency of the DSME content. Given the extant research that shows Black men with diabetes have significant barriers to health care, we offer DSME with the assumption that most participants have not received formal DSME.

Next, participants will transition into 6 90-min weekly PL led DSMS sessions intentionally designed for older Black men with T2D. A core component of PLDSMS is for PLs to provide DSMS with the oversight of the CDCES. While the CDCES will not be present in the PL-led DSMS, they will meet with the CDCES once each month to answer questions and help plan for the next DSMS session(s) as needed. In prior work, we observed that PLs were most effective and confident when they had ongoing support to maximize their efforts in the areas of clinical content, educational methods, and group facilitation and communication skills. The patient-directed, DSMS session content is organized around 6 core processes [28]. 1) reflecting on relevant self-management experiences, 2) discussing emotions, 3) problem-solving barriers to diabetes management, 4) addressing questions about diabetes, 5) setting behavioral goals and 6) patient-provider communication strategies. PLs will refer any clinical questions that are raised by participants to the CDCES. During DSME, participants will also be given the culturally specific and literacy-appropriate guidebook, “Lifelong Diabetes Self-Management Guidebook,” which was developed for previous projects and was well received [22]. Peer Leader DSMS will be delivered in a group format, via the virtual platform Zoom [42] for Health at U-M, with two groups of 15 participants each. Each group will be assigned two PLs.

##### Control group (Control *n* = 30)

Participants randomized to the control group will receive 6 sessions of group-delivered DSME provided by a CDCES; however they will not receive any DSMS or ongoing support from PLs and the PL will not participate in the DSME sessions. The primary purpose of the trial is to evaluate recruitment and retention rates, intervention satisfaction and estimate intervention effect sizes. As such, the EUC condition was chosen as the control in order to 1) ensure that any intervention effects are not due to provision of diabetes education alone, 2) minimize ethical concerns regarding assignment of underserved populations to receive a no-treatment control and 3) control for improvements due to attention and positive regard and expectancies for improvement due to participation in treatment (i.e. Hawthorne effects).

##### Peer leaders (*n* = 4)

Participants recruited to be PLs will attend PL training (described below), co-facilitate DSME sessions with a CDCES, lead DSMS sessions, and complete the same battery of assessments as all other participants in the study. To ensure PLs are supported, monthly meetings will be held with all PLs so that they may exchange information and support each other. PLs will be compensated at $10/h but only through the conclusion of DSMS (T2). This payment schedule mimics the real-world scenario of when the research grant is over. A toolkit will be provided containing: 1) DSMS training curriculum and manuals 2) data collection instruments and databases to allow for continuous program evaluation and 3) detailed information on the functions staff need to play to sustain improvements in outcomes.

### Outcomes

#### Primary outcome measures

Self-management behaviors will be measured using the Perceived Diabetes Self-Management Scale, a self-report questionnaire used to measure a broad range of management behaviors, such as insulin management, dietary management, blood glucose monitoring, symptom response, and parent assistance/supervision.

Metabolic Control will be measured via hemoglobin A1C (A1C). A1C will be collected using the DCA 2000 point-of-care testing instrument. The DCA hematology analyzer performs hemoglobin A1C and microalbumin/creatinine tests.

#### Secondary outcome measures

Adherence to gender norms will be measured using the Male Role Norms Inventory-Short Form. BMI will be calculated using height and weight. Height will be measured using a stadiometer. Weight will be measured on a high quality, calibrated digital scale. BP will be measured using the auscultatory method.

Diabetes Social Support will be measured using the Diabetes Social Support Questionnaire. Diabetes-related Distress will be measured using Diabetes Distress Scale (SF-12) and lastly, a validated Diabetes Quality of Life will be used to measure quality of life. Participants will also complete questionnaires that assess socio-demographic, behavioral, psychosocial, and health services utilization and are validated in diverse populations with diabetes.

### Participant timeline

Data will be collected at baseline (T0), completion of DSME and Peer Led DSMS (T1, 3 months, approximate treatment termination) and follow-up (T3, 3 months post intervention) (See Table 1).

**Table 1:** Detailed data collection schedule and measurement items

TIMEPOINT (state unit)				
**VISIT NUMBER:**	-T1	T0	T1	T2
	Screening, Enrollment, Week 1	Baseline assessment, Allocation, Week 2	3-month Intervention close-out	3-Month Post-Intervention Follow-up
**ENROLLMENT:**				
Eligibility screen	X			
Informed consent	X			
Allocation		X		
**INTERVENTIONS:**				
CDCES-Led Diabetes Self-Management Education + Peer Led Diabetes Self-Management Support		X	X	X
Control Group		X	X	X
**ASSESSMENTS:**				
Biological/anthropometric measures		X	X	X
Demographic/background information questionnaire		X	X	X
Perceived Diabetes Self-Management Scale		X	X	X
Adherence to gender norms-Male Role Norms Inventory-Short Form		X	X	X
Diabetes Social Support Questionnaire		X	X	X
Diabetes Distress Scale		X	X	X
Diabetes Quality of Life Scale		X	X	X

### Sample size

#### Power analysis

Assuming 20% attrition, we expect a final sample size of 48 (excluding the four participants designated as peer leaders), approximately 12 per group (with 2 groups in the treatment arm). If we assume correlations of 0.25 between successive measurements of A1C, then this sample size will yield power of 0.8 to detect a difference of 0.6 standard deviation between average values of A1C in treatment and control groups.

### Participant recruitment

We will recruit through the Michigan Center for African American Aging Research Participant Resource Pool (MCUAAAR PRP) community-partners. The MCUAAAR PRP is a research volunteer registry can be accessed by scholars conducting research of Black makes, 55 years of age and older who meet their study criteria. Dr. Hawkins has previously conducted studies using older Black males with diabetes recruited from the PRP. There are currently a total of 1424 active PRP members and 60.1% of male PRP members have diabetes. Interested men will call a central office phone number where they will be scheduled for screening. Men with diabetes identified with the help of the Pepper Center and the MCUAAAR PRP will be called by study staff and invited to participate in the intervention.

### Allocation

Participants in this unblinded study will be randomly allocated to one of the two groups based on block randomization. A random number sequence generated by a computer will be created, which will then be used in the randomization feature in Research Electronic Data Capture (REDCap).

After an individual provides informed consent at the baseline visit (visit 1), the participant will be asked to return for visit 2 1 week later. Prior to visit 2, a study coordinator without prior knowledge of the sequence will randomize the participant via REDCap, allocate the individual to the selected group, and inform the participant of their assigned group at visit 2. Participants will be informed of their assigned group at the second visit in attempt to maximize the likelihood of continuation in the study.

### Blinding (masking)

Blinding throughout the duration of the study is not feasible because the notification of each earned incentive is part of the intervention.

### Data collection

#### Trial procedures and evaluations

##### Peer leader recruitment, training and assessment

Peer leaders will be recruited from the Phase 1 interviews and/or identified by senior center staff. Peers for Progress suggests using community linkages and social networks, and/or asking community agencies for nominations of their clients to serve as peer leaders. Once identified, PLs will receive 30 h of training to be conducted over 3 months. To decrease burden on PLs the trainings will be spread out over 3 months and scheduled with the input of PLs for convenience. The PL training curriculum will be based on materials used by Piatt et al. [37] who successfully trained 19 PLs with a 100% attendance rate. Training will be group-based and include both the knowledge and skills needed to implement empowerment-based DSMS. A CDCES who was involved in the development and implementation of the training curriculum in previous projects, will conduct the training. A description of the content and skill areas that will be covered as part of the training can be found elsewhere [28]. Formative (during training) assessment of skills will consist primarily of observing skills and giving feedback. Summative (end of training) assessment includes written assessments to evaluate concepts and all required skills. Peer leaders will complete post-training measures that assess empowerment-based facilitation, communication skills and goal setting and must achieve: 1) mean of ≥4 on 5-point Likert scales on the Understanding Management Practice (UMP) 94 2) empowerment-based facilitation across 6 video vignettes 3) the Active Listening Observation Scale-global scale [43] and 4) the Diabetes Empowerment Scale Short-Form [44]. Three attempts will be given to successfully complete the training. As noted above, it is expected that this content will be adapted to include discussion of men’s health concerns based on Phase 1 with a focus on beliefs that affect men’s health and modeling of alternative perspectives that allow for healthy behaviors to be framed as competence and strength.

##### Data collection procedures

The PI and co-investigators will train all research staff in standardized data collection techniques and measurement. All quantitative and qualitative will be collected at baseline (T0), 3 months (T1), 6 months (T2), (estimated length of data collection: 60 min at each time point). All physiologic data collection will be conducted at the senior center to increase convenience for participants. Self-report questionnaires will be completed telephonically or virtually and were developed in a previous study [36]. Participants will be compensated $20 for completing each assessment. Men who withdraw will still be asked to participate in study data collection visits. Data will be stored in REDCap, a secure, web-based application hosted at UM (see Data Management).

##### Instruments and measures. Primary outcomes

*Glycemic control* will be measured with hemoglobin A1C (A1C) and collected using the DCA 2000 point-of-care testing instrument in order to maintain consistency of measurement over time. *Self-management* will be measured using the Perceived Diabetes Self-Management Scale, a self-report questionnaire used to measure a broad range of management behaviors, such as insulin management, dietary management, blood glucose monitoring, and symptom response.

##### Secondary outcomes

Secondary outcomes include depression**,** Body Mass Index (BMI), Blood pressure, Diabetes Social Support, Diabetes-related Distress, the SF-12, Diabetes Quality of Life and adherence to gender norms. Other variables include socio-demographic, behavioral, psychosocial, and health services utilization measures [45–57].

#### Retention

Our research team has substantial experience with retention of high-risk samples that are similar in nature to the proposed sample. The following procedures will be used to minimize participant attrition: 1) Data collection sessions will be completed at the community center, telephonically or online in order to maximize the convenience of data collection for subjects and 2) multiple techniques are used to increase the likelihood that participants will keep their data collection appointments, including advanced scheduling, multiple reminder letters and phone reminders.

### Data management

At the time of study entry, participants will be assigned a Global Unique Identifier (GUID) to be used on all study materials and data for the duration of the study. Confidentiality is of prime importance. Anonymity will be assured to all research participants so that no participants will be named in any reports about the study data. All data collected will be used only for research purposes. The Dr. Hawkins will retain control of all data collected, including questionnaires, audiotapes, and transcribed notes. Data will be stored in a locked file drawer and in a locked office that is accessible only to the PI and study personnel.

### Statistical methods

#### Outcomes

##### Phase 1

Qualitative analyses will be conducted during Phase 1 and on the post-intervention interviews. Interviews will be recorded and transcribed. We will develop codes utilizing a grounded theory approach and will start with the formulation of categories and definitions developed directly from the text. Through this process a coding a manual and definitions will be finalized. The refined manual will be used to guide ongoing coding and pairs of coders will read subsequent transcripts keeping codes that achieve 80% agreement on code application. Analyses will be conducted using Atlas.ti. For Phase 1, these data will be used to conduct a final refinement of treatment content as needed. Post-intervention, intervention components from the perspective of participants will be assessed using interviews to establish comprehension, acceptability and feasibility of the intervention and to detect any domain-specific issues.

##### Phase 2

The proposed study includes a sample size of 64 Black men, 30 in the intervention arm, 30 in the control arm and 4 trained as peer leaders for the intervention arm. Assuming a 20% overall attrition rate (which is a conservative estimate given that our research groups have only experienced 5–10% attrition in prior clinical trials with Black samples recruited in the Detroit area), we anticipate a final analyzed sample of 48 (excluding peer leaders). This will result in 24 participants in each group, which is consistent with sample sizes typically adopted in pilot intervention trials. A goal of the revised intervention is to increase retention rates of Black men in the PLDSMS intervention. Based on our past studies’ intervention retention rates (90–95%), a PLDSMS intervention retention rate of 75% will be considered sufficient to proceed to the R01. We plan to power a future R01 using the adapted PLDSMS intervention based on a minimally clinical importance difference (e.g., 0.5% decrease in A1C) rather than based on specific effect sizes from this trial. Nevertheless, a finding that the intervention resulted in a small effect on the primary and secondary endpoints would suggest the need to conduct further refinement activities with the intervention prior to proceeding to a fully powered trial. In order to evaluate our ability to disseminate findings from the current study and detect between group differences, we will assess statistical significance for the intervention group by time interaction using an Individually Randomized Group-Treatment Trial, with intervention group as a between-subjects factor (2 levels), repeated measurements over time as a within subjects factor (3 levels), a within-subject correlation of .5, and an α of .05, an a non-sphericity correction of .75. The effect size used in this design is Cohen’s f (f = σmeans/σ). The effect of the intervention on diabetes management and A1C (primary outcomes) will be assessed using mixed-effects models. Diabetes management and A1C at baseline, post intervention, and follow-up will be used as the dependent variables. Independent variables include intervention group, time of assessment, and interaction between time and intervention group. To take into account correlation between observations, random intercepts and slopes will be included into the model. In the analysis we will follow intention-to-treat principles. Analyses will be conducted with adjusting for stratifying variables (i.e. age group).

### Data monitoring

#### Formal committee

To ensure the proper monitoring of the safety of all participants and the quality of data collected, a Data Safety and Monitoring Board (DSMB) will be established that will follow techniques suggested in the literature [58]. The board will consist of the PI and study team members. In addition, five outside members, who are not involved with the study, will serve on the DSMB as voting members and will be identified at a later date. Voting members will consist of University professors with experience and expertise in clinical diabetes intervention research to ensure consistency and quality of input.

A Critical Incident Journal will be maintained throughout the study of the proposed intervention to document the nature of any adverse events, involvement of personnel, interventions considered, and interventions implemented to address the event. When necessary the research team will consult with the Human Research Protection Program (HRPP) Internal Review Board housed in the University of Michigan Office of Research at University of Michigan. All research team members have completed human subjects protection certifications at their home institutions.

Data integrity will be reviewed by the research team in conjunction with the project coordinator and in consultation with the Data Safety and Monitoring Panel. Data from the intervention will be entered into a Microsoft Access database. The research team will meet every 3 months to discuss issues related to recruitment, implementation of the intervention, participant safety, and to monitor data trends (e.g., adherence, missing data).

### Safety/harms

Due to the nature and objective of the study, participants will be asked to provide finger stick capillary blood samples during assessments to measure A1C levels. Risks associated with finger stick capillary blood draws include: minor discomfort from obtaining the blood sample, minor pain, bruising, or bleeding at the puncture site similar to any other routine blood sample collections.

At each assessment period, participants will be asked to complete a series of psychosocial questionnaires. Completion of the Beck Depression Inventory, the demographic questionnaire, the Diabetes Quality of Life measure, the SF-36 and others may cause minor discomfort for participants. Participants will be informed that they may discontinue completion of individual items, questionnaires, or the study protocol at any time. Participants who experience significant discomfort may meet with the Principal Investigator. Risk associated with venipuncture will be mitigated by the use of skilled personnel experienced in blood draws and sample collection.

Finally, with self-report surveys, there is also the small risk that prompting patients to review their diabetes care practices and providing them with feedback about their diabetes-related health outcomes (e.g., A1C, blood pressure) could cause some emotional discomfort or anxiety. Such discomfort would likely prime patients and their primary care physician or group facilitator to address any problems identified. Other risks include breach of confidentiality of study data.

Research staff will be trained in research ethics, confidentiality protection, and HIPAA prior to and throughout the study period through the Human Research Protection Program (HRPP) in the University of Michigan Office of Research (UMOR). ho will maintain participant confidentiality about the content of group discussions.

All questionnaires and instrumentations are standardized measures that have been used in our own trials and in other diabetes research and there are no significant risks anticipated related to the completion of them. However, breaks will be given as needed to reduce fatigue, and research assistants will be appropriately trained to obtain personal information in a sensitive fashion. Research staff will be trained in research ethics, confidentiality protection, and HIPAA prior to and throughout the study period. All peer leaders must pass standardized training prior to providing service to participants and will also be trained in protection of participant confidentiality.

At the time of study enrollment, participants will be assigned a study identification number to be used in all study materials and data for the duration of the study. All identifying information will be separated from the data and laboratory values. A master list that contains participants names and study identification number will be kept in a locked filing cabinet in the School of Social Work. Audiotapes of interviews and exit interviews will also be stored securely on a password protected computer only accessible to study personnel and will be destroyed upon study completion. The corresponding principal investigator (Dr. Hawkins) and the research assistant will be the only persons who have access to the file linking study ID# to each subject. Participants will be assured that all data they provide to the study will be confidential unless it is necessary to “alert” both the patient and their physician because of a laboratory value outside of the normal ranges that reflects a risk requiring immediate attention. All reports will use aggregate data. Subject names or other identifiers will not be reported. No persons from the Detroit-based senior center will handle or have access to personal health information or participant survey data. Throughout the intervention period, study participants will have access to the phone number of the project coordinator in the event of any adverse events. The Principal Investigator and Co-Investigators will be available by phone to speak to participants in this event.

### Auditing

To ensure treatment fidelity, all peer-led DSMS sessions will be recorded and rated using fidelity checklists developed by our research team.

## Discussion

Diabetes is a chronic illness that requires ongoing, sustained self-management education and support that is patient-driven, and flexible to the dynamic and evolving condition of patients’ “real-world” environment and life circumstance [3]. Our current health care system and professional work force is insufficient to meet the needs of our patient population with diabetes. If successful, this proposed project will improve the health of a high-risk population in need of more effective diabetes management strategies.

In light of the rates of type 2 diabetes in African American men and the considerable costs associated with sub-optimally managed diabetes, interdisciplinary community-based diabetes intervention effectiveness trials are needed. To date, the diabetes intervention literature is both compelling and efficacious in the predominantly white, middle class, urban samples in which it has been tested. Additionally, for diabetes interventions focused on African American populations, the sample sizes are often a majority female [11]. Few of these intervention protocols have been translated to underserved populations in urban settings with a focus on African American men [11]. Moreover, prior diabetes trials have not made full use of existing community members (peer leaders) to test the feasibility and sustainability of diabetes self-management support for African American men [12, 13]. Additionally, no studies have taken advantage of gender-matching for lay helpers for DSMS with older African American men with type 2 diabetes a model that has proven useful in prevention and harm reduction work being done with African American men [12, 13]. Taken together, peer-led DSMS interventions, existing community resources and tailoring to the needs of African American men with diabetes can have great potential to work synergistically to improve diabetes outcomes through enhancement of metabolic control.

Data from this study will contribute to the expansion of the diabetes intervention literature by contributing the experience of African American men in an urban setting using an interdisciplinary treatment approach. Data from this study will also contribute to the literature on translational research with underserved urban patients with diabetes. Finally, data from this study may be disseminated and used by community organizations to adapt and adopt community-based interventions that will promote the health of patients with diabetes.

## Data Availability

Not applicable.

## Abbreviations

DSMES: Diabetes self-management education and support
DSMS: Diabetes self-management support
DSME: Diabetes self-management education
T2D: Type 2 diabetes
UM IRB: University of Michigan institutional review board
HRPP: Human research protection program
UMOR: University of Michigan office of research
HIPAA: Health insurance portability and accountability act of 1996
DSMB: Data safety and monitoring board
GUID: Global unique identifier
CDCES: Certified diabetes care and education specialists
PL: Peer leader
RCT: Randomized clinical trial
TDF: Theoretical domains framework
